# 30th German Cancer Congress, 22nd–25th February 2012, Berlin

**DOI:** 10.3332/ecancer.2012.251

**Published:** 2012-03-22

**Authors:** L Budiner

**Affiliations:** GNS Press –International News Agency, Ingolstadt, Germany

## Abstract

“Securing quality – calling for and encouraging research”.

From 22nd to 25th February 2012 one of the largest multi-disciplinary cancer congresses took place in Berlin, Germany, attended by more than 10,000 healthcare professionals. “The German Cancer Congress is the only interdisciplinary congress of its kind in Germany and the second largest congress of its kind anywhere in the world” stated the congress president Professor Dr P Albers and the president of the German Cancer Society (DKG), Professor Dr Dr h.c. W Hohenberger in their greetings. Only the ASCO Annual Meeting is larger. At the 30th German Cancer Congress (DKK) established national and international experts presented latest data and discussed hot topics in the various oncology fields. Joint Symposia of the DKG together with the UICC (Union for International Cancer Control) and ASCO (American Society of Clinical Oncology) respectively stressed the importance of the meeting. The overreaching theme of the meeting was “Securing quality – calling for and encouraging research”. This theme evolved from the idea, that only excellent research in clinics and medical practices can found the basis of future high quality efficient and effective structures in patient care and of the allocation of limited resources to meaningful measures for patient benefit.

## Changing the Political Landscape

Political topics dominated the German Cancer Congress and a focus of discussions at the joint sessions with ASCO and UICC. Key questions posed were: How to advance and encourage research in an environment with limited resources? How can we ensure high quality of cancer care and treatment taking into account the limited resources and the increasing cancer incidence? All questions of worldwide relevance.

Douglas Blayney, Medical Director of the Stanford Cancer Institute, Palo Alto (California), chaired the ASCO and DKG joint symposium entitled “Quality of Care” and introduced the QOPI (Quality Oncology Practice Initiative) organised by the American Society of Clinical Oncology. “Currently the participating practices routinely measure the quality of their care,” but Blayney announced, that “…the next phase of quality improvement will focus on outcomes of care, in addition to the processes of care.” Both Blayney and the current meeting president, Peter Albers, who co-chaired the joint meeting, underlined their interest in a cooperation in order to synergise to improve quality of care in the US and Germany. Other topics treated at the joint meeting were the development and implementation of guidelines, the certification of cancer centres and the relevance of cancer registries. Independent from these activities some promising initiatives to ensure quality of care and data transparency are already in place in Germany, e.g. the Western-German Breast Cancer Centre (WBC, http://www.doc-holding.de) with detailed benchmarking reports.

The joint UICC and DKG international workshop on “Health Care Structures” was chaired by U Kleeberg, chairman of section A of the DKG, and E L Cazap, president of the UICC. The workshop addressed complimentary medicine as well as political aspects of health care and epidemiological data from Latin America and Germany. Interestingly the cancers causing frequent death in the two regions populations differed largely. In Chile e.g. the second most common cancer causing death in women is gall bladder cancer, as J Gallardo from Santiago de Chile mentioned “It could be supportive to investigate reasons for these differences in cancer mortality in order to recognise new risk factors”. Within single Latin American countries there is a large diversity in population genetics and traditions. Obesity as one major risk factor for cancer is increasing in many countries. Cancer prevention including healthy food and moderate sport is difficult to implement. In many countries, there is only limited access to new treatment options and appropriate treatment. The differences between public and private health care are extremely high and experts tend to migrate from the public to the private sector for economic reasons. This migration causes a lack of high level experts in the public sector and thus results in poorer quality of care there. Supporting projects of the UICC could help to improve the situation of patients with cancer and raise awareness of the disease worldwide (for detailed information see www.uicc.org).

New challenges in cancer care are emerging. The number of cancer survivors will increase due to the demographic changes and better therapeutic options. In Germany there is now a considerable number of cancer survivors – currently about 1.7 million – living with a chronic cancer disease.

In an aging society with increasing cancer incidence and survival, limits to research, cancer care and quality of care are defined by restricted financial and human resources. In order to maintain a high quality cancer care and cancer research in the future, resources have to be allocated carefully and based on a long-term planning. A well-conceived, well-managed national cancer plan allows proper allocation of resources, facilitates early cancer detection and improves the life of patients with cancer. Therefore the German National Cancer Plan (GNCP) was re-launched shortly before the Cancer Congress and discussed there in detail. The meeting program reflected the key topics of the GNCP leaning on the following pillars and considerations:

1. Cancer screening/early detection
  - Special focus of the GNCP given to cervical and colon cancer.
  - Further screening programs are available for breast and prostate cancer as well as for melanoma and others are under development.
  - The sensitivity of the screening methods is still an issue because of “over-diagnosed” (false positive) patients. One approach to reduce this over screening in patients with breast cancer could be a risk-adapted screening protocol aiming at defined, well-known risk groups who should be screened frequently whereas other low-risk groups (with many false positive diagnoses) might be screened later or with less frequency. Peter Albers explained this approach from his experience with prostate cancer screening. Future research will have to focus on this topic.
2. Further development of cancer care structures and quality management
  - Systematic data processing is the key to reliable data.
  - New IT-networking/communication tools are needed to generate reliable data of today’s cancer treatment and tomorrow’s patient outcome in order to shape future efficient and effective patient pathways and health care processes. In Germany a partnership between health insurances, health care professionals (or their organisations like the DKG) and IT – providers is necessary to find the most convenient solutions and prepare for e-health. In fact this topic was the subject under discussion at an evening meeting, accompanying the congress. The congress president, P Albers, together with the executive director of the DKG, J Bruns, the head of pathology at one of Europe’s leading hospitals, the Charité Hospital, Berlin M Dietel, and the president of the German Cancer Society W Hohenberger discussed at length the future of e-health with the CEO of Deutsche Telekom (a leading telecommunication company) R Obermann, and the leader of the health care division within the Deutsche Telekom, A Wehmeier. They agreed on developing in close collaboration between providers and users, e.g. practice based and hospital based oncologic health care professionals and the “Deutsche Telekom”, new tools for e-health. Furthermore concerted action plans should also raise awareness in politicians and society in order to prepare the legal basis for e-health.
  - Systematic data evaluation in cancer registries is the basis for cancer research and will be in the focus over the next years in Germany and Europe. “Also in the European context, cancer registries should be mandatory in order to get detailed European cancer data” – mentioned Nils Wilking, from the Karolinska Institute, Stockholm. Profound knowledge of cancer epidemiology, regional differences in incidence and treatment and patient outcome could open new perspectives in cancer research. In a country like Germany, a nation-wide cancer registration was implemented only some years ago and there are still some regions without a proper registry.
  - Further investment in the implementation of certified cancer centres and Comprehensive Cancer Centres (CCC) to improve cancer care and research structures are planned in Germany. Currently the German Cancer Research Centre (DKFZ, Deutsches Krebsforschungszentrum) one out of eleven CCC’s is organising a translational research network in Germany.
  - Personal communication skills within the interdisciplinary cancer care team have to be properly developed. Training programmes will be prepared and implemented.
  - Implementation of evidence-based guidelines is the key to good treatment and optimised patient outcome. In Germany the so called S3 evidence-based guidelines for diagnoses and treatment of different cancers (e.g. breast and colon cancer) are available and will be developed further. They are publicly available at www.awmf.de.
3. Assurance of efficient oncological treatment (currently emphasising on pharmacotherapy as basis for an evidence based medicine)
  - New approaches to post-launch drug evaluations must be defined and optimised.
  - The generation of data concerning best drug dosing, optimal sequence, side effects and their management etc. is crucial.
  - The structures and the health care system have to be prepared for personalised medicine, including molecular pathology.
4. Patient empowerment (patient focus and information)
  - Training programmes should aim to improve communication skills both for patients and their physicians. Patients should be trained in order to better understand informed consents with regard to their treatment.
  - Psycho-Oncologic care should be available and offered early to patients.
  - Patients should be more actively involved in research and health care system related decision-making.
  - Palliative care should aim to allow patients to maintain the maximum of quality of life until the end of their lives.

## Shaping the scientific landscape

Beside the political topics, focus of the congress lay on science. The funding and prioritisation of research in oncology was discussed broadly with key investigators and funding organisations including the Ministry of Science and Research and the Ministry of Health. They agreed that improved management of limited resources for research is crucial in order to keep pace with global research activities. An aligned action plan for the different private and governmental funding organisations is required and a clearer focus and structure in research is mandatory. But so far no actions have been taken.

Also as human resources are limited, it is increasingly important to encourage young people to work in the demanding but highly advanced area of oncology. Especially for this target group the Junior Academy and the Forum for Young Science at the DKK 2012 was set up with the aim of inspiring the interest of young scientists in this area.

In plenary sessions emphasis was given to palliative care in key tumour areas such as skin, prostate, colorectal, breast and lung as well as leukaemia’s and lymphomas. In “State of the Art” sessions current concepts of diagnosis and treatment were discussed. Special focus was dedicated to “Cancer of the elderly” and “Long-Term-Survivorship” as these are topics which will need more attention as cancer survivorship is extended. In the “Best-of” sessions selected presentations of the best posters were given. Some of the major highlights are listed here [1].

## Skin Cancer Highlights

Best Poster (#B5-0178): “Apoptosis induction in melanoma cells and reduced melanoma growth in mice by oncolytic adenoviral vectors armed with TRAIL” presented by L F Fecker [2], Skin Cancer Centre of the Charité Hospital, Berlin. TRAIL = TNF-related apoptosis inducing ligand. Basis for the considerations were the selective expression of E1A in melanoma cells. This led to the construction of oncolytic AdVs with death ligands and inducible expression of TRAIL or CD 95L. Properties of AdV-TRAIL/AdV-CD95L are selective replication in melanoma cells and selective oncolysis, as well as selective/adjustable induction of apoptosis. AdV-TRAIL showed a reduction of tumour growth in mice. The antitumour activity is enhanced by chemotherapeutics.

Best Poster (# B6-0275): “Cytotoxicity of new duplex drugs linking 3′-c-ethynylcytidine and 5-fluor-2′-deoxyuridine against human melanoma cells” presented by S Schott [3], National Cancer Institute, Heidelberg. Since no cure exists for metastatic disease (stage IV), there is an urgent need for novel drugs to overcome melanoma therapy resistance. New drugs offer new therapeutic options in melanoma, but resistance occurs frequently. Duplex drugs might help to prolong therapy and could be added to the sequence to overcome resistance. Both drugs showed meaningful effects and should be investigated further.

Best Poster (#B7-0332): “Inhibition of the PI3K-AKT signalling pathway to overcome therapy resistance in melanoma-derived brain metastasis” by H Niessner *et al* [4], University Hospital Tübingen. Brain metastases occur in over 70% of patients with metastatic melanoma and are often subject to therapy resistance. The activation of the RAF-MEK-ERK and PI3K-AKT-mTOR signal transduction pathways makes a decisive contribution to tumour progression and treatment resistance. Niessner *et al* asked: “Could the blockade of the RAF-MEK-ERK or/and PI3K-AKT-mTOR signalling pathways be a promising strategy for the treatment of melanoma brain metastases?” The results of their research suggest that activation of AKT is relevant for the survival and growth of melanoma cells in the brain parenchyma and that inhibition of PI3K-AKT signalling may be a suitable strategy to enhance and/or prolong the antitumor effect of BRAF inhibitors in melanoma brain metastases.

## Lung Cancer Highlights

Best Poster (# B12 – 0475): “Prognostic value of oestrogen (ESR-1) receptor expression in curatively resected non-small cell lung cancer (NSCLC)” by Brueckl *et al* [5], Clinic Nuremberg, Department of Internal Medicine. Despite undergoing complete resection of NSCLC, 33% and 77% of patients with stage IA and IIIA, respectively, die within 5 years. Brueckl already presented first data on his research in metastatic NSCLC on ASCO 2011 annual meeting. He was able to show better outcome in oestrogen (ESR-1) receptor expressing tumours in the palliative treatment of metastatic NSCLC (Brueckl [6] *et al*, Proceedings of ASCO 2011). The main objective of this study was to test, whether ESR-1 expression is of prognostic/predictive value in the curative setting as well. It was shown that ESR-1 expression is of positive prognostic value in curatively resected NSCLC. Patients with ESR-1 low/non expressing tumours might benefit most from adjuvant chemotherapies with the hormone receptor status being of predictive value. However, these later data are preliminary and warrant further confirmation. The oestrogen receptor status is a well-established marker in breast cancer, frequently used to take decisions about endocrine treatment. In NSCLC, it is not usual to determine the status of these receptors. ESR-1 is a positive prognostic marker and should be investigated further.

[Personal comment on this findings from the author: higher mortality of lung cancer patients with hormone replacement therapy was observed in 2006 by Ganti *et al* [7] – are there any links to ESR-1 expression?]

Best Poster (#B9-0198): “Hypofractionated image-guided breath-hold radiotherapy of pulmonary tumours and metastases” – clinical results presented by A Frauenfeld [8], University Medical Centre, Mannheim. A unique treatment technology for inoperable lung cancer was presented. Despite the unfavourable patient selection, high local control rates could be achieved. If a reasonably high BED2 can be applied, image-guided breath-hold SBRT (Stereotactic Ablative RadioTherapy) is an effective non-invasive treatment modality with a high local control rate and relatively low toxicity in patients with inoperable lung tumours and lung metastases. As disease progression was mainly outside the treated area, systemic therapy has to be improved.

## Endometrial Cancer Highlights

Best Poster (# B17- 0067): The German study group for gynaecologic oncology (AGO = arbeitsgemeinschaft gynäkologische onkologie) represented by G Emons [9], University Hospital Goettingen, presented its results to the Phase II-study with a targeted cytotoxic LHRH-analogue (AEZS-108) in patients with LHRH – receptor positive endometrial cancer: protocol AGO-GYN 5, AGO-study group. Endometrial cancers (EC) commonly express receptors for luteinising hormone releasing hormone (LHRH). AEZS-108 is a targeted cytotoxic drug where [D-Lys(6)]-LHRH is linked to doxorubicin. Efficacy and toxicity of AEZS-108 was assessed in endometrial cancer. A promising clinical benefit rate of 72% was shown. OS after single agent AEZS-108 is similar to that reported for modern triple combination chemotherapy, but was achieved with distinctly lower toxicity.

## Molecular Pathology Highlights

New predictive testing was presented by Denkert [10], Department of Pathology, Charité Hospital, Berlin, at the session for oncology pathology (AOP) at the DKK 2012. The test is developed for the application in patients with breast cancer (C Denkert *et al*, Virchows Arch 2012, in press). The test called “EndoPredict” allows predicting whether a hormone receptor positive and Her2 negative breast cancer patient would benefit from a single endocrine treatment. Further investigation for molecular pathology in Berlin is ongoing and focused on ovarian cancer based on TOC (Tumour Bank Ovarian Cancer) housed by the Charité Hospital and built with tissue input from four European countries.

## Head and Neck Cancer Highlights

Squamous cell carcinomas of the head and neck are in general on the decline, the impact of classical risk factors decline as well, while the association with oncogenic papillomavirus infections, in particular in cancers of the oropharynx, is increasing considerably. J P Klußmann [11] discussed the importance of human papilloma viruses in carcinoma of the oropharynx. Tumours of the head and neck are either HPV positive or negative, but the incidence of HPV positive carcinomas is increasing, especially in young men. The biology and pathology of HPV positive and negative tumours is different and the treatment might differ including targeted therapies in the future. He suggests future vaccination of men against HPV and showed his own cases and data of the University Hospital Gießen-Marburg. Klußmann is part of the AHMO – the workgroup for head and neck cancers.

## Glioblastoma Highlights

Glioblastoma in elderly –NOA8 study of the Neurooncologic working group was introduced by W Wick *et al* [12]. Details of the NOA8 cannot be presented, as the data have been submitted and accepted by ASCO. Wick explained, that elderly are often not sufficiently treated. There is a trend towards under-treatment in elderly patients with cancer.

## Rectal Cancer Highlights

Two linked clinical trials were presented. The German multicentre phase III trial CAO/ARO/AIO-94 compared preoperative chemoradiotherapy (CRT) with postoperative CRT in locally advanced rectal cancer. Preoperative CRT was found to improve local control and was associated with reduced acute toxicity but did not improve survival. At the DKK 2012 Merkel [13] (# 131 and 133) and Roedel [14] presented long-term outcome data of this trial. Even after a median follow-up of 11 years, preoperative CRT remained superior to postoperative CRT with respect to locoregional recurrences. The indication for multimodal treatment has to be based on evidence-based criteria, because long-term survivors complain about reduced quality of life, especially with respect to bowel function and defecation. However, long-term quality of life was found to be superior after preoperative CRT when compared to postoperative CRT. Fietkau, Erlangen, presented on behalf of R Sauer [15] the preliminary results of the randomised phase III trial CAO/ARO/AIO-04 on “Preoperative chemoradiotherapy and postoperative chemotherapy with 5-FU and oxaliplatin versus 5-FU alone in locally advanced rectal cancer”. Trial design was based on the outcome of the first a.m. trial. The primary endpoint DFS cannot yet be reported as the data are not mature enough. Fietkau underlined, that with 5-FU/oxaliplatin and also with 5-FU alone compliance was comparable. No major differences could be observed so far in both arms. Slightly improved pCR without increasing toxicity was observed in the oxaliplatin-containing arm.

The German Cancer Society awarded this year’s prize to three well-known scientists. The prize covers three areas of research: clinical, experimental and translational. This year the prize was granted to:

- Professor Dr Michael Bamberg, Department of Radio-oncology, University Hospital Tübingen, for his clinical research with regard to cancer of the brain.
- Professor Dr Florian Greten, Department of Intern Medicine, University Hospital Munich, for experimental studies concerning the molecular mechanisms responsible for colon cancer. The results of his studies open new perspectives for research in colon cancer therapies.
- Professor Dr Charlotte Niemeyer, Department of Paediatric Oncology and Haematology, University Hospital Freiburg, for her translational research concerning bone marrow diseases preceding leukaemias. Her research enhanced the understanding of the mechanisms responsible for the development of myelodyplastic syndromes.
